# Calcium ion regulation by BAPTA-AM and ruthenium red improved the fertilisation capacity and developmental ability of vitrified bovine oocytes

**DOI:** 10.1038/s41598-017-10907-9

**Published:** 2017-09-06

**Authors:** Na Wang, Hai-Sheng Hao, Chong-Yang Li, Ya-Han Zhao, Hao-Yu Wang, Chang-Liang Yan, Wei-Hua Du, Dong Wang, Yan Liu, Yun-Wei Pang, Hua-Bin Zhu, Xue-Ming Zhao

**Affiliations:** 1 Embryo Biotechnology and Reproduction Laboratory, Institute of Animal Sciences (IAS), Chinese Academy of Agricultural Sciences (CAAS), No. 2 Yuanmingyuan Western Road, Haidian District, Beijing, 100193 China; 2 Livestock and Poultry Import & Export Dept, China Animal Husbandry Group (CAHG), No. 188 West Road, South 4th Ring Road, Beijing, 100070 China

## Abstract

Vitrification reduces the fertilisation capacity and developmental ability of mammalian oocytes; this effect is closely associated with an abnormal increase of cytoplasmic free calcium ions ([Ca^2+^]i). However, little information about the mechanism by which vitrification increases [Ca^2+^]i levels or a procedure to regulate [Ca^2+^]i levels in these oocytes is available. Vitrified bovine oocytes were used to analyse the effect of vitrification on [Ca^2+^]i, endoplasmic reticulum Ca^2+^ (ER Ca^2+^), and mitochondrial Ca^2+^ (mCa^2+^) levels. Our results showed that vitrification, especially with dimethyl sulfoxide (DMSO), can induce ER Ca^2+^ release into the cytoplasm, consequently increasing the [Ca^2+^]i and mCa^2+^ levels. Supplementing the cells with 10 μM 1,2-bis (o-aminophenoxy)ethane-N,N,N′,N′-tetraacetic acid (BAPTA-AM or BAPTA) significantly decreased the [Ca^2+^]i level and maintained the normal distribution of cortical granules in the vitrified bovine oocytes, increasing their fertilisation ability and cleavage rate after *in vitro* fertilisation (IVF). Treating vitrified bovine oocytes with 1 μM ruthenium red (RR) significantly inhibited the Ca^2+^ flux from the cytoplasm into mitochondria; maintained normal mCa^2+^ levels, mitochondrial membrane potential, and ATP content; and inhibited apoptosis. Treating vitrified oocytes with a combination of BAPTA and RR significantly improved embryo development and quality after IVF.

## Introduction

Cryopreservation of oocytes plays an important role in providing oocytes for assisted reproductive technologies, including *in vitro* fertilisation (IVF), intracytoplasmic sperm injection, and somatic cell nuclear transfer^1–3^. Oocyte cryopreservation also contributes to infertility treatment in humans by avoiding ethical and legal problems of human embryo freezing^4^. Currently, slow freezing and vitrification are two methods used for oocyte cryopreservation; of these, vitrification is considered to be better^5–7^ because the high concentration of cryoprotectants (CPAs) used and the extremely high cooling rates help to prevent the formation of ice crystals^4, 8^.

Meanwhile, vitrification decreases the fertilisation ability and developmental competence of oocytes^3, 9–12^, which greatly limits its wide application in embryonic biotechnology. This phenomenon is closely associated with the abnormal increase of cytoplasmic free calcium ions ([Ca^2+^]i) in vitrified oocytes^9^, which then triggers the premature release of cortical granules (CGs) to the zona pellucida (ZP) layers^13, 14^, resulting in abnormal ZP hardening before fertilisation. However, how vitrification increases the [Ca^2+^]i level in oocytes remains unclear.

Endoplasmic reticulum (ER) and mitochondria are important Ca^2+^ pools in oocytes^15^, and the [Ca^2+^]i increase at fertilisation is primarily derived from the ER^15, 16^. Mitochondria are responsible for Ca^2+^ absorption and release and play an important role in the conduction of [Ca^2+^]i signalling^17^. Until now, the effect of vitrification on ER Ca^2+^ and mitochondrial Ca^2+^ (mCa^2+^) levels, which would help to determine the mechanism by which vitrification increases [Ca^2+^]i level in oocytes, has not yet been fully investigated.

The Ca^2+^ chelator 1,2-bis(o-aminophenoxy)ethane-N,N,Nʹ,Nʹ-tetraacetic acid (BAPTA-AM or BAPTA) significantly reduces the level of [Ca^2+^]i^18, 19^. The mCa^2+^ uniporter ruthenium red (RR) can inhibit the influx of Ca^2+^ into the mitochondria^20^. A previous study reported that 10 μM BAPTA treatment obviously reduced ZP hardening in vitrified mouse oocytes^9^; another study reported that thawed vitrified porcine germinal vesicle oocytes treated with 1 μM RR or 10 μM BAPTA showed significantly improved survival and maturation rates^21^. However, little information is available regarding the mechanism by which RR and BAPTA can improve the development ability of vitrified oocytes.

Therefore, in the present study, we investigated the effect of vitrification on [Ca^2+^]i, ER Ca^2+^, and mCa^2+^ levels in vitrified bovine oocytes to determine the mechanism by which the [Ca^2+^]i level is increased in vitrified bovine oocytes. Based on our findings, we further investigated the effect of BAPTA and RR on the [Ca^2+^]i, ER Ca^2+^, and mCa^2+^ levels; CG distribution; mitochondrial function; apoptosis; and fertilisation ability of vitrified bovine oocytes to illustrate the means through which BAPTA and RR improve the developmental ability of vitrified oocytes.

## Results

In our study, 7015 of 7702 (91.1 ± 6.2%) MII oocytes survived after vitrification.

### Experiment 1: Effect of vitrification on Ca^2+^ levels in bovine oocytes

**Figure 1:**
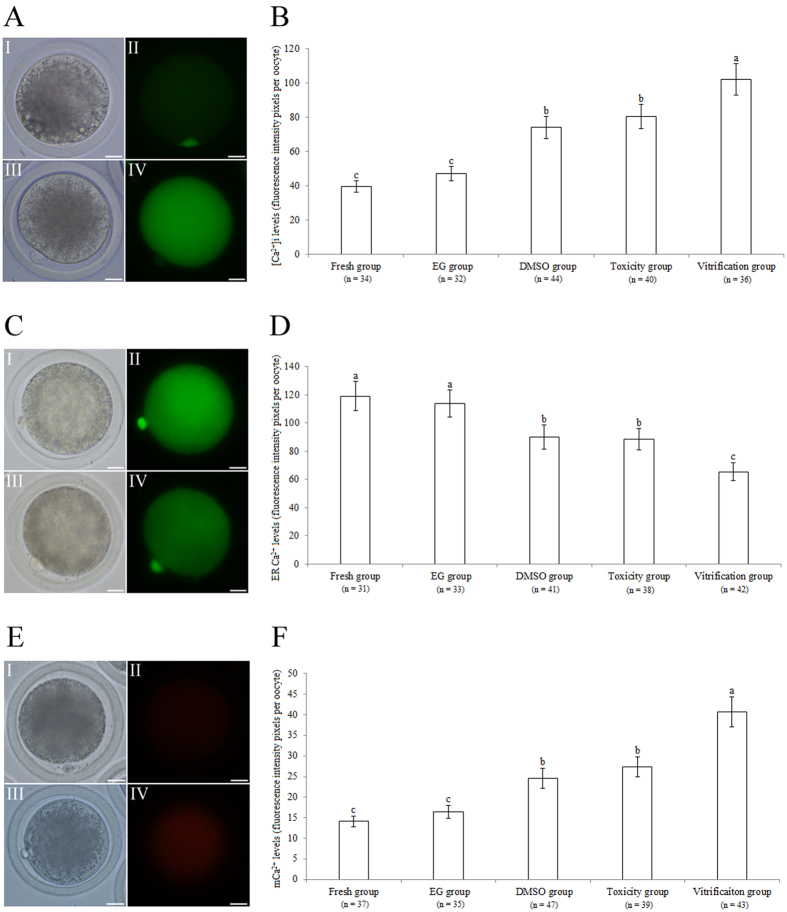
Effect of vitrification on the Ca^2+^ levels of bovine oocytes. (**A**,**C**,**E**) Bovine oocytes stained with [Ca^2+^]i, ER Ca^2+^, and mCa^2+^ detection kits. (I, II) oocytes from the fresh group. (III, IV) oocytes from the vitrification group. Scale bar = 20 μm. (**B**,**D**,**F**) [Ca^2+^]i, ER Ca^2+^, and mCa^2+^ levels in bovine oocytes, respectively. ^a,b,c^: Values with different superscripts differ significantly between groups (*P* < 0.05).

Figure 1A illustrates the [Ca^2+^]i staining of bovine oocytes. As shown in Fig. 1B, the [Ca^2+^]i level was significantly higher in the vitrification group than in the ethylene glycol (EG), dimethyl sulfoxide (DMSO), toxicity, and fresh groups (*P* < 0.05). The [Ca^2+^]i levels of the DMSO and toxicity groups were significantly higher than those of the EG and fresh groups (*P* < 0.05). The [Ca^2+^]i levels did not significantly differ between the EG and fresh groups (*P* > 0.05).

Figure 1C shows the ER Ca^2+^ staining of bovine oocytes. As shown in Fig. 1D, the ER Ca^2+^ level was significantly lower in the vitrification group than in the EG, DMSO, toxicity, and fresh groups (*P* < 0.05). The ER Ca^2+^ levels in the DMSO and toxicity groups were similar and were both significantly lower than those in the EG and fresh groups (*P* < 0.05). No significant difference was found in the ER Ca^2+^ levels between the EG and fresh groups (*P* > 0.05).

Figure 1E illustrates the mCa^2+^ staining of bovine oocytes. As shown in Fig. 1F, the mCa^2+^ level was significantly higher in the vitrification group than in the EG, DMSO, toxicity, and fresh groups (*P* < 0.05). The mCa^2+^ levels in the DMSO and toxicity groups were similar and were significantly higher than the levels in the EG and fresh groups (*P* < 0.05). No significant difference was found in mCa^2+^ levels between the EG and fresh groups (*P* > 0.05).

### Experiment 2: Effect of BAPTA treatment on Ca^2+^ levels and developmental ability of vitrified bovine oocytes

**Figure 2:**
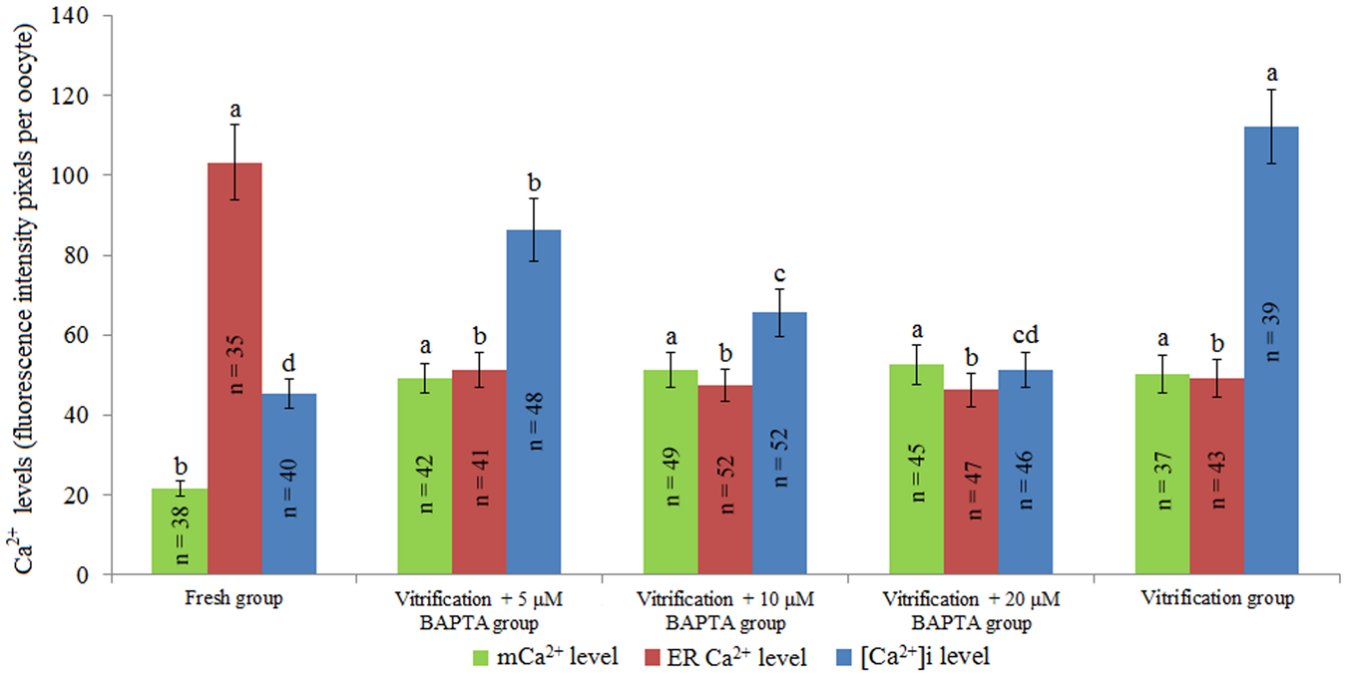
Effect of BAPTA treatment on Ca^2+^ levels of vitrified bovine oocytes. ^a,b,c,d^Values with different superscripts differ significantly between groups (*P* < 0.05).

As shown in Fig. 2, the [Ca^2+^]i level of the vitrification + 10 μM BAPTA group was significantly lower than that of the vitrification + 5 μM BAPTA group and vitrification group (*P* < 0.05) but still significantly higher than that of the fresh group. Meanwhile, the [Ca^2+^]i level of the vitrification + 20 μM BAPTA group was similar to that of the fresh group. The mCa^2+^ levels did not significantly differ between the three vitrification + BAPTA groups (5, 10, and 20 μM) and the vitrification group (*P* > 0.05), and these levels were all significantly higher than that of the fresh group (*P* < 0.05). The ER Ca^2+^ levels of the three vitrification + BAPTA groups (5, 10, and 20 μM) were similar to that of the vitrification group (*P* > 0.05) and were all significantly lower than that of the fresh group (*P* < 0.05).

Fig. S1 shows the CG staining of bovine oocytes. As shown in Table 1, the percentage of oocytes with peripheral distribution of CGs in the vitrification + 20 μM BAPTA group (63.5 ± 6.0%) was significantly higher than that in the vitrification + 5 μM, vitrification + 10 μM BAPTA and vitrification groups (39.3 ± 3.6%, 52.2 ± 4.8%, and 25.0 ± 2.2%, respectively; P < 0.05) and similar to that of the fresh group (70.6 ± 6.3%; P > 0.05). The percentages of oocytes with complete release and discontinuous peripheral distribution in the vitrification + 5 μM BAPTA (30.4 ± 2.7%, 19.6 ± 1.9%), vitrification + 10 μM BAPTA (19.4 ± 1.6%, 17.9 ± 1.0%), and vitrification + 20 μM BAPTA (11.1 ± 1.0%, 15.9 ± 1.4%) groups were significantly lower than the corresponding values in the vitrification group (39.1 ± 3.3%, 23.4 ± 2.1%; P < 0.05) but still higher than those in the fresh group (7.8 ± 0.6%, 11.8 ± 1.3%; P < 0.05). There was no significant difference in the percentages of oocytes with a homogeneous distribution of CGs among all groups (P > 0.05; Table 1).

**Table 1:** Effect of BAPTA treatment on the CG distribution in vitrified bovine oocytes.

Groups	Number of oocytes	Number of oocytes with peripheral distribution	Number of oocytes with discontinuous peripheral distribution	Number of oocytes with complete release of CGs	Number of oocytes with homogeneous distribution
Vitrification group	64	16 (25.0 ± 2.2%)^d^	15 (23.4 ± 2.1%)^a^	25 (39.1 ± 3.3%)^a^	8 (12.5 ± 1.1%)^a^
Vitrification + 5 μM BAPTA group	56	22 (39.3 ± 3.6%)^c^	11 (19.6 ± 1.9%)^b^	17 (30.4 ± 2.7%)^b^	6 (10.7 ± 0.9%)^a^
Vitrification + 10 μM BAPTA group	67	35 (52.2 ± 4.8%)^b^	12 (17.9 ± 1.0%)^bc^	13 (19.4 ± 1.6%)^c^	7 (10.5 ± 0.9%)^a^
Vitrification + 20 μM BAPTA group	63	40 (63.5 ± 6.0%)^a^	10 (15.9 ± 1.4%)^c^	7 (11.1 ± 1.0%)^d^	6 (9.5 ± 0.6%)^a^
Fresh group	51	36 (70.6 ± 6.3%)^a^	6 (11.8 ± 1.3%)^d^	4 (7.8 ± 0.6%)^e^	5 (9.8 ± 0.8%)^a^

^a,b,c,d,e^Values with different superscripts differ significantly within the same column (*P* < 0.05).

Fig. S2 illustrates the fertilisation capacity of bovine oocytes after IVF (Supplementary Methods 1.6). As shown in Table 2, the percentage of monospermic oocytes in the vitrification + 10 μM BAPTA group (61.0 ± 5.8%) was significantly higher than that in the vitrification group (42.1 ± 4.0%; P < 0.05) and similar to that in the vitrification + 5 μM BAPTA (51.6 ± 4.3%), vitrification + 20 μM BAPTA (65.2 ± 6.0%), and fresh groups (69.8 ± 6.3%; P > 0.05). The percentages of polyspermic and unfertilised oocytes of the vitrification + 10 μM BAPTA group (15.3 ± 1.4%, 23.7 ± 2.3%) were significantly lower than those of the vitrification group (23.4 ± 1.9%, 34.6 ± 3.2%; P < 0.05) and similar to those of the vitrification + 20 μM BAPTA group (11.4 ± 0.7%, 21.4 ± 1.9%) and fresh group (10.5 ± 1.0%, 19.8 ± 1.7%; P > 0.05).

**Table 2:** Effect of BAPTA treatment on the fertilisation capacity of vitrified bovine oocytes.

Groups	Number of oocytes	Number of polyspermic oocytes	Number of monospermic oocytes	Number of unfertilised oocytes
Vitrification group	107	25 (23.4 ± 1.9%)^a^	45 (42.1 ± 4.0%)^c^	37 (34.6 ± 3.2%)^a^
Vitrification + 5 μM BAPTA group	95	19 (20.0 ± 1.8%)^ab^	49 (51.6 ± 4.3%)^bc^	27 (28.4 ± 2.2%)^b^
Vitrification + 10 μM BAPTA group	118	18 (15.3 ± 1.4%)^bc^	72 (61.0 ± 5.8%)^ab^	28 (23.7 ± 2.3%)^c^
Vitrification + 20 μM BAPTA group	112	15 (11.4 ± 0.7%)^c^	73 (65.2 ± 6.0%)^a^	24 (21.4 ± 1.9%)^c^
Fresh group	86	9 (10.5 ± 1.0%)^c^	60 (69.8 ± 6.3%)^a^	17 (19.8 ± 1.7%)^c^

^a,b,c^Values with different superscripts differ significantly within the same column (*P < *0.05).

As shown in Table 3, the cleavage rate of the vitrification + 10 μM BAPTA group (65.7 ± 5.7%) was significantly higher than those of the vitrification (41.6 ± 3.8%) and vitrification + 20 μM BAPTA (49.7 ± 4.3%; *P* < 0.05) groups and similar to those of the vitrification + 5 μM BAPTA group (52.5 ± 4.2%) and fresh group (75.0 ± 6.2%; *P* > 0.05). For the blastocyst rate, no significant difference was found among the vitrification + 5 μM BAPTA (15.1 ± 1.4%), vitrification + 10 μM BAPTA (15.7 ± 1.2%), vitrification + 20 μM BAPTA (12.0 ± 0.9%), and vitrification (12.4 ± 1.1%; *P* > 0.05) groups, and they were all significantly lower than that of the fresh group (34.1 ± 2.8%; *P* < 0.05).

**Table 3:** Effect of BAPTA treatment on the developmental ability of vitrified bovine oocytes after IVF.

Groups	Number of oocytes	Number of cleaved oocytes (%)	Number of blastocysts (%)
Vitrification group	233	97 (41.6 ± 3.8%)^c^	12 (12.4 ± 1.1%)^b^
Vitrification + 5 μM BAPTA group	177	93 (52.5 ± 4.2%)^bc^	14 (15.1 ± 1.4%)^b^
Vitrification + 10 μM BAPTA group	213	140 (65.7 ± 5.7%)^ab^	22 (15.7 ± 1.2%)^b^
Vitrification + 20 μM BAPTA group	185	92 (49.7 ± 4.3%)^c^	11 (12.0 ± 0.9%)^b^
Fresh group	176	132 (75.0 ± 6.2%)^a^	45 (34.1 ± 2.8%)^a^

^a,b,c^Values with different superscripts differ significantly within the same column (*P* < 0.05).

### Experiment 3: Effect of RR treatment on Ca^2+^ levels and developmental potential of vitrified bovine oocytes

**Figure 3:**
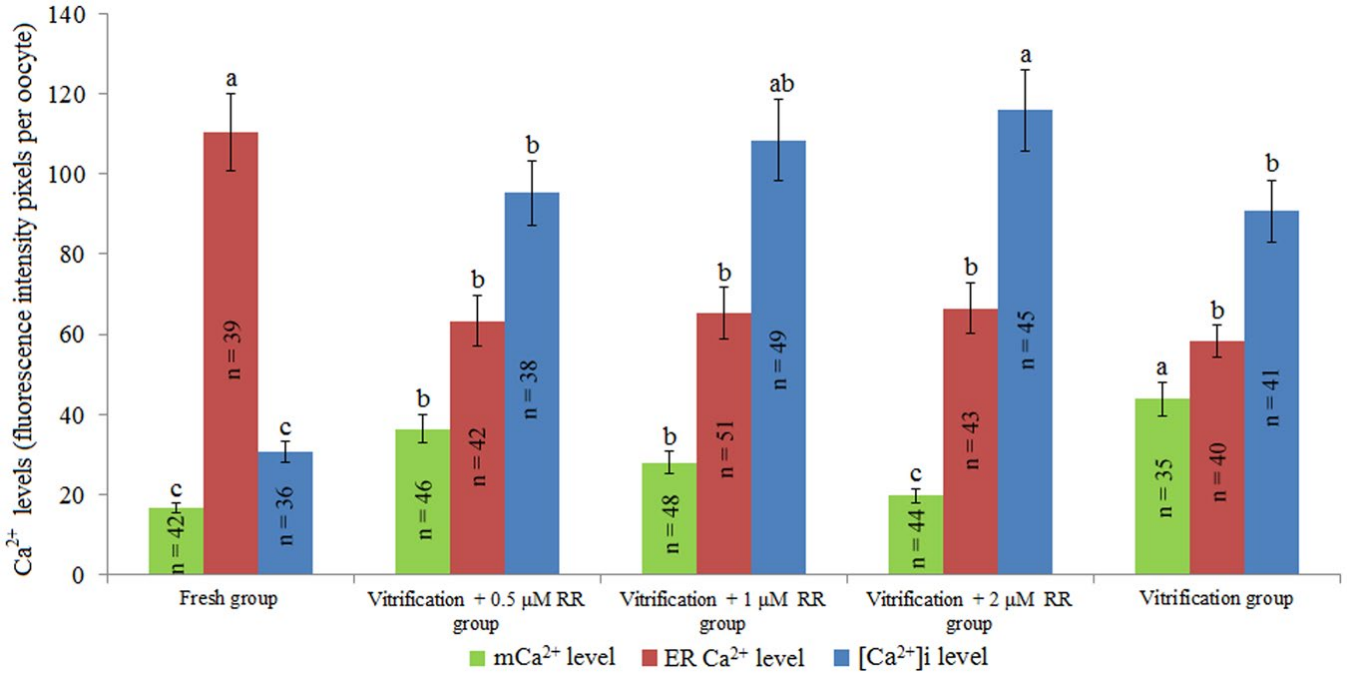
Effect of RR treatment on Ca^2+^ levels in vitrified bovine oocytes. ^a,b,c^Values with different superscripts differ significantly between groups (*P* < 0.05).

As shown in Fig. 3, the mCa^2+^ levels were significantly lower in the vitrification + RR (0.5, 1, and 2 μM) groups than in the vitrification group (*P* < 0.05). The mCa^2+^ levels were similar between the vitrification + 0.5 μM RR group and vitrification + 1 μM RR group, and these levels were significantly higher than those of the fresh and vitrification + 2 μM RR groups (*P* < 0.05). No statistically significant difference was observed in mCa^2+^ levels between the vitrification + 2 μM RR group and fresh group (*P* > 0.05).

The ER Ca^2+^ level of the vitrification + 0.5 μM RR group was similar to those of the vitrification + 1 μM RR group, vitrification + 2 μM RR group, and vitrification group but significantly lower than that of the fresh group (*P* < 0.05). The [Ca^2+^]i level of the vitrification + 2 μM RR group was similar to that of vitrification + 1 μM RR group, and this value was significantly higher than those of the 0.5 μM RR group, vitrification group, and fresh group (*P* < 0.05).

Figure S3 illustrates the mitochondrial membrane potential (∆Ψm) of bovine oocytes stained with JC-1, and Fig. S4 illustrates terminal deoxynucleotidyl transferase-mediated dUTP nick-end labelling (TUNEL)-stained bovine oocytes. As shown in Table 4, the ration of red to green fluorescence intensity and ATP content of the vitrification + 0.5 μM RR group (1.3 ± 0.1, 0.8 ± 0.1 pmol), vitrification + 1 μM RR group (1.7 ± 0.1, 1.0 ± 0.1 pmol), and vitrification + 2 μM RR group (1.4 ± 0.1, 0.8 ± 0.1 pmol) were significantly higher than those of the vitrification group (0.9 ± 0.1, 0.6 ± 0.0 pmol; P < 0.05). However, TUNEL-positive oocyte percentages in the vitrification + 0.5 μM RR group (16.7 ± 1.1%), vitrification + 1 μM RR group (8.2 ± 0.7%), and vitrification + 2 μM RR group (17.8 ± 1.1%) were significantly lower than that in the vitrification group (31.4 ± 2.7%; P < 0.05). The ratio of red to green fluorescence intensity, ATP content, and percentage of TUNEL-positive oocytes were all similar between the vitrification + 1 μM RR group (1.7 ± 0.1, 1.0 ± 0.1 pmol, 8.2 ± 0.7%) and fresh group (1.7 ± 0.2, 1.1 ± 0.1 pmol, 8.3 ± 0.6%; P > 0.05).

**Table 4:** Effect of RR treatment on the ratio of red to green fluorescence intensity, ATP content, and apoptosis of vitrified bovine oocytes.

Groups	Ratio of red to green fluorescence in oocytes	ATP content (pmol per oocyte)	Percentage of TUNEL-positive oocytes
Vitrification group	0.9 ± 0.1^c^ (n = 35)	0.6 ± 0.0^c^ (n = 40)	31.4 ± 2.7%^a^ (n = 51)
Vitrification + 0.5 μM RR group	1.3 ± 0.1^b^ (n = 41)	0.8 ± 0.1^b^ (n = 40)	16.7 ± 1.1%^b^ (n = 42)
Vitrification + 1 μM RR group	1.7 ± 0.1^a^ (n = 45)	1.0 ± 0.1^a^ (n = 40)	8.2 ± 0.7%^c^ (n = 49)
Vitrification + 2 μM RR group	1.4 ± 0.1^b^ (n = 42)	0.8 ± 0.1^b^ (n = 40)	17.8 ± 1.1%^b^ (n = 45)
Fresh group	1.7 ± 0.2^a^ (n = 37)	1.1 ± 0.1^a^ (n = 40)	8.3 ± 0.6%^c^ (n = 36)

^a,b,c^Values with different superscripts differ significantly within the same column (*P* < 0.05).

As shown in Table 5, cleavage and blastocyst rates were significantly lower in the vitrification group (66.0 ± 5.7%, 22.9 ± 1.4%) than in the fresh group (82.1 ± 4.6%, 43.7 ± 4.1%; *P* < 0.05), and no significant difference was found between the vitrification + 0.5 μM RR group (72.9 ± 6.9%, 28.1 ± 2.4%) and vitrification group (66.0 ± 5.7%, 22.9 ± 1.4%; *P* > 0.05). However, the cleavage and blastocyst rates of the vitrification + 1 μM RR group (84.0 ± 7.2%, 39.7 ± 3.9%) were significantly higher than those of the vitrification group (66.0 ± 5.7%, 22.9 ± 1.4%; *P* < 0.05) and similar to those of the fresh group (82.1 ± 4.6%, 43.7 ± 4.1%; *P* > 0.05).

**Table 5:** Effect of RR treatment on the development potential of vitrified bovine oocytes after PA.

Groups	Number of oocytes	Number of cleaved oocytes (%)	Number of blastocysts (%)	Total cell number
Vitrification group	212	140 (66.0 ± 5.7%)^b^	32 (22.9 ± 1.4%)^c^	57.4 ± 4.9^b^ (n = 30)
Vitrification + 0.5 μM RR group	210	153 (72.9 ± 6.9%)^ab^	43 (28.1 ± 2.4%)^bc^	64.6 ± 5.2^b^ (n = 30)
Vitrification + 1 μM RR group	225	189 (84.0 ± 7.2%)^a^	75 (39.7 ± 3.9%)^a^	75.8 ± 6.6^a^ (n = 30)
Vitrification + 2 μM RR group	183	133 (72.7 ± 5.2%)^ab^	45 (33.8 ± 2.3%)^ab^	62.4 ± 4.6^b^ (n = 30)
Fresh group	173	142 (82.1 ± 4.6%)^a^	62 (43.7 ± 4.1%)^a^	82.6 ± 7.7^a^ (n = 30)

^a,b,c^Values with different superscripts differ significantly within the same column (*P* < 0.05).

The total blastocyst cell number of the vitrification + 0.5 μM RR group (64.6 ± 5.2) was similar to that of the vitrification + 2 μM RR (62.4 ± 4.6) and vitrification (57.4 ± 4.9; *P* > 0.05) groups and significantly lower than that in the fresh group (82.6 ± 7.7; *P* < 0.05). However, the total blastocyst cell number in the vitrification + 1 μM RR group (75.8 ± 6.6) was significantly higher than that in the vitrification group (57.4 ± 4.9; *P* < 0.05) and similar to that in the fresh group (82.6 ± 7.7; *P* > 0.05).

### Experiment 4: Effect of BAPTA and RR on the developmental ability of vitrified bovine oocytes after IVF

**Figure 4:**
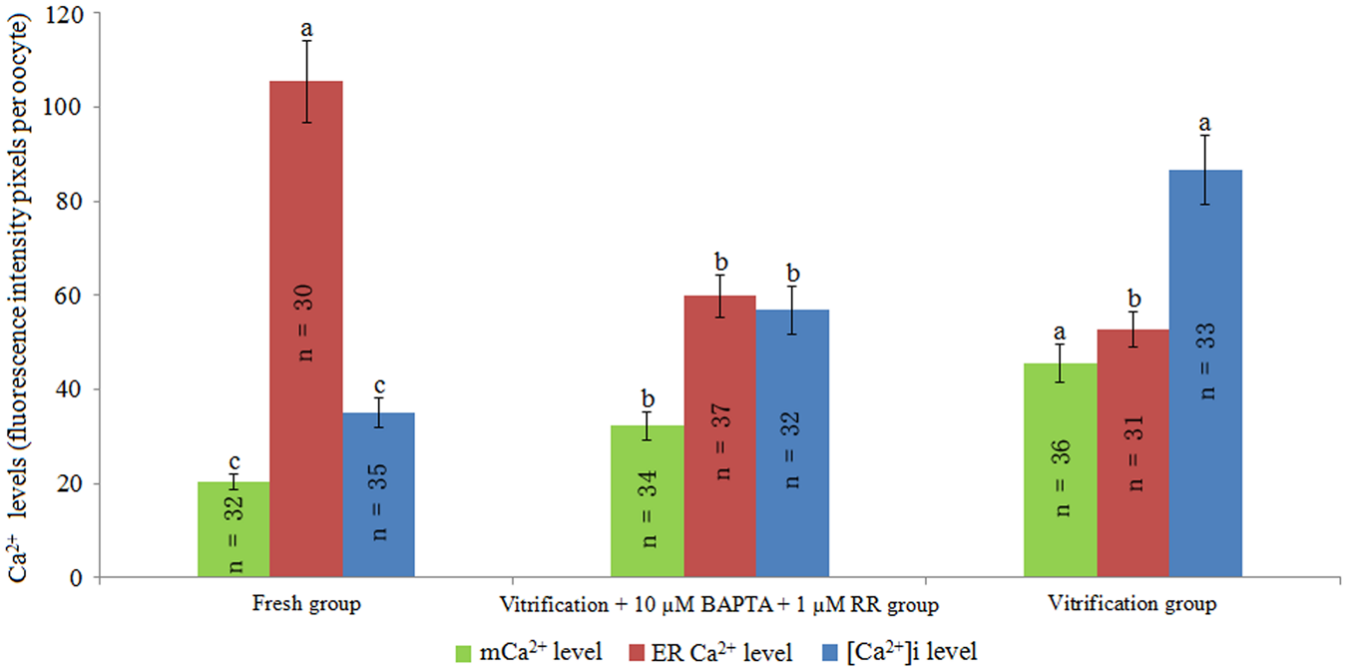
Effect of BAPTA + RR on Ca^2+^ levels in vitrified bovine oocytes. ^a,b,c^Values with different superscripts differ significantly between groups (*P* < 0.05).

As shown in Fig. 4, the mCa^2+^ and [Ca^2+^]i levels of the vitrification + 10 μM BAPTA + 1 μM RR group were significantly lower than those of the vitrification group (*P* < 0.05) but still higher than those of the fresh group. Meanwhile, the ER Ca^2+^ level of the vitrification + 10 μM BAPTA + 1 μM RR group was similar to that of the vitrification group and lower than that of the fresh group (*P* < 0.05).

Fig. S5 illustrates blastocysts stained with Hoechst-33342. As shown in Table 6, the percentages of cleaved oocytes and blastocysts and total cell number per blastocyst were higher in the vitrification + 10 μM BAPTA + 1 μM RR group (74.4 ± 5.2%, 30.1 ± 2.7%, 99.7 ± 8.4) than in the vitrification group (51.6 ± 4.4%, 10.4 ± 0.9%, 83.1 ± 6.4; P < 0.05); the values in the vitrification + 10 μM BAPTA + 1 μM RR group were similar to those in the fresh group (80.7 ± 7.8%, 34.4 ± 2.3%, 103.8 ± 9.6; P > 0.05).

**Table 6:** Effect of BAPTA + RR treatment on the development of vitrified bovine oocytes after IVF.

Groups	Number of oocytes	Number of cleaved oocytes (%)	Number of blastocysts (%)	Total cell number
Vitrification group	896	462 (51.6 ± 4.4%)^b^	48 (10.4 ± 0.9%)^b^	83.1 ± 6.4^b^ (n = 30)
Vitrification + 10 μM BAPTA + 1 μM RR group	648	482 (74.4 ± 5.2%)^a^	145 (30.1 ± 2.7%)^a^	99.7 ± 8.4^a^ (n = 30)
Fresh group	612	494 (80.7 ± 7.8%)^a^	170 (34.4 ± 2.3%)^a^	103.8 ± 9.6^a^ (n = 30)

^a,b^Values with different superscripts differ significantly within the same column (*P* < 0.05).

**Figure 5:**
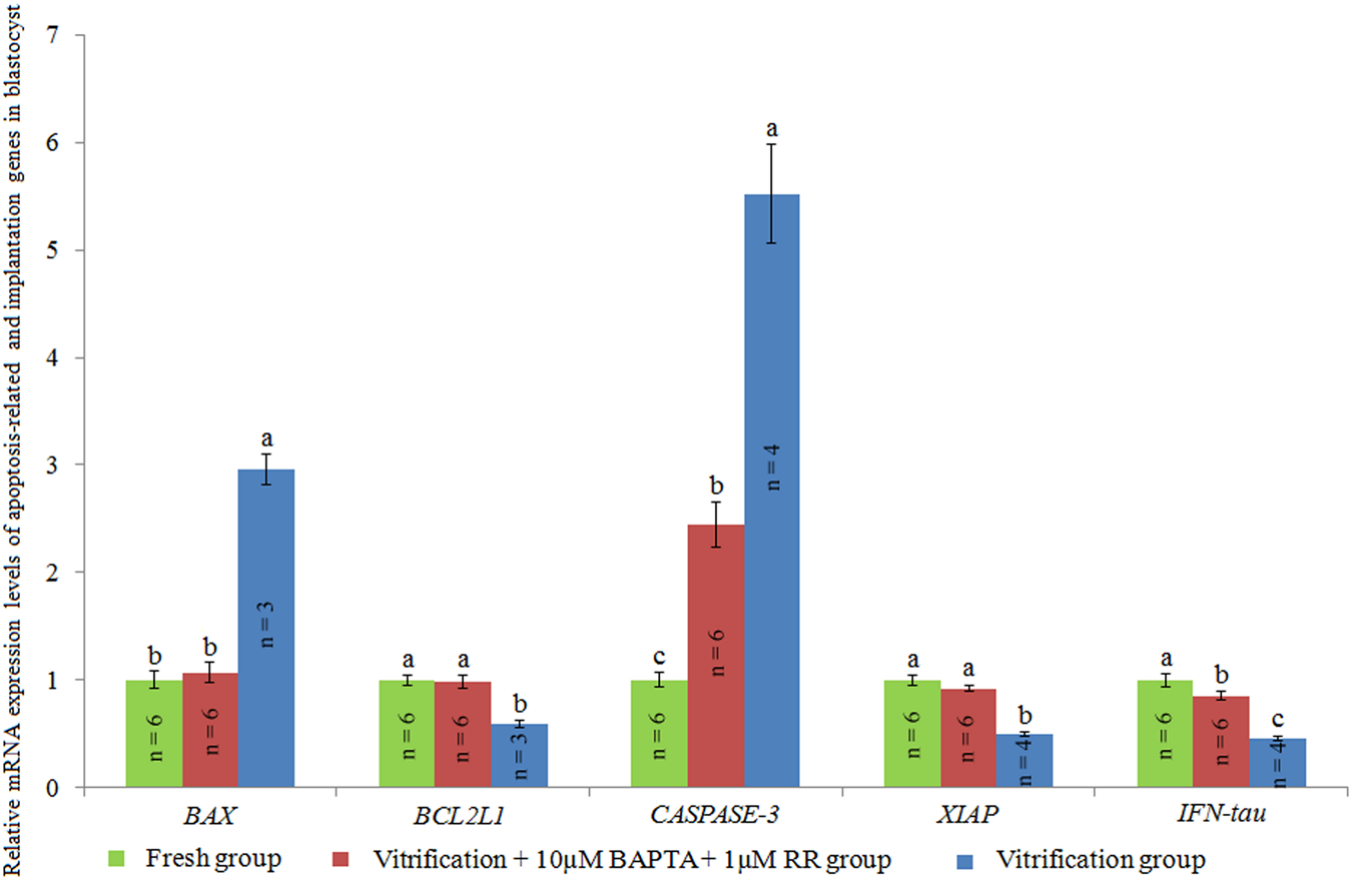
Effect of BAPTA + RR on the mRNA expression levels of apoptosis-related and implantation genes in blastocysts. ^a,b,c^Values with different superscripts differ significantly between groups (*P* < 0.05).

As shown in Fig. 5, the mRNA expression levels of anti-apoptotic genes *BCL2L1* and *XIAP* and the pregnancy recognition signal gene *IFN-tau* were significantly higher in blastocysts of the vitrification + 10 μM BAPTA + 1 μM RR group than in those of the vitrification group, while the mRNA expression levels of pro-apoptosis genes *BAX* and *CASPASE-3* were lower in blastocysts of the vitrification + 10 μM BAPTA + 1 μM RR group than in those of the vitrification group.

## Discussion

EG is a widely used CPA in vitrification solution; it causes Ca^2+^ influx across the plasma membrane from the oocyte culture medium, which results in an increase in the [Ca^2+^]i level^9, 22^. Therefore, DPBS without Ca^2+^ was used as the base medium to prepare vitrification solution in the present study to relieve Ca^2+^ influx from the medium into the cytoplasm of oocytes. In the present study, vitrification was found to significantly increase the [Ca^2+^]i level in bovine oocytes (Fig. 1B); this finding is similar to previous results reported in murine^9, 22, 23^ and feline^24^ oocytes. As we used DPBS without Ca^2+^ to prepare vitrification solution in our study, the increase in the [Ca^2+^]i level may be partially due to Ca^2+^ release induced by vitrification from internal Ca^2+^ pools in oocytes. Furthermore, our results showed that DMSO, and not EG, induced the increase in the [Ca^2+^]i level in vitrified oocytes, indicating that DMSO increases [Ca^2+^]i by triggering Ca^2+^ release from the internal Ca^2+^ pools of oocytes, which confirmed the previous results of Larman *et al*.^9, 22^.

As shown in Fig. 1D, our experiment demonstrated that vitrification significantly decreased the ER Ca^2+^ level in bovine oocytes. Furthermore, our results proved that it was DMSO, and not EG, that induced Ca^2+^ release from ER in vitrified oocytes, which was due to the nonspecific effect of DMSO on the ER membrane. DMSO also triggered premature Ca^2+^ release from the ER^9^. Interestingly, our experiment showed that vitrification significantly increased the mCa^2+^ level in bovine oocytes (Fig. 1F). Theoretically, the mCa^2+^ level in vitrified oocytes should be reduced as DMSO has a nonspecific effect on the mitochondrial membrane, inducing the release of mCa^2+^ ^9^. The increased mCa^2+^ level in vitrified oocytes was due to Ca^2+^ influx from the ER to the cytoplasm, which promotes Ca^2+^ flow into the mitochondria^25, 26^. Taken together with these results, our experiments provided strong evidence showing that vitrification, especially with DMSO, significantly induced Ca^2+^ release from the ER, leading to abnormal increases of [Ca^2+^]i and mCa^2+^ in oocytes.

As shown in Fig. 2, our results showed that BAPTA did not influence mCa^2+^ or ER Ca^2+^ levels, but it significantly decreased [Ca^2+^]i levels in vitrified bovine oocytes because BAPTA is an effective chelator of [Ca^2+^]i^27^. During oocyte maturation, CGs translocate from the cytoplasm to the adjacent plasma membrane and represent the maturation of the cytoplasm^28, 29^. As shown in Table 1, our results showed that vitrification significantly decreased the percentage of oocytes with a peripheral distribution of CGs and increased the percentages of oocytes with discontinuous distribution and completed release of CGs. This effect is similar to a phenomenon observed previously in vitrified ovine oocytes^30^ and human oocytes^31, 32^. These results may be due to the increased [Ca^2+^]i level in vitrified bovine oocytes, which can trigger the release of CGs to the cytoplasm^28, 33^. Meanwhile, the percentage of vitrified oocytes with peripheral distribution of CGs was significantly increased in all three BAPTA groups because BAPTA is capable of decreasing the [Ca^2+^]i levels of vitrified oocytes.

With regard to fertilisation capacity, our results showed that vitrification significantly decreased the percentage of monospermic oocytes and thus increased the percentages of polyspermic and unfertilised oocytes (Table 2), and this result was closely associated with a lower percentage of oocytes with peripheral distribution of CGs in the vitrification group, as it is necessary for prevention of polyspermy^28, 33^. Meanwhile, the higher percentage of vitrified oocytes with peripheral distribution of CGs could partly explain why the 10 µM BAPTA group had a higher percentage of monospermic and cleaved oocytes after IVF (Tables 2 and 3). However, treatment with BAPTA only was not sufficient to improve blastocyst development from vitrified oocytes (Table 3).

As shown in Fig. 3, our results demonstrated that RR treatment significantly decreased the mCa^2+^ level in vitrified bovine oocytes because RR blocks the influx of increased [Ca^2+^]i into the mitochondria of vitrified oocytes^20^. ΔΨm and ATP content are indicators of mitochondrial function^34, 35^. Similar to previous results reported for bovine^36–38^, mouse^39^ and porcine^40^ oocytes, our results showed that ΔΨm was significantly decreased in bovine oocytes by vitrification (Table 4). Our experiment also revealed that vitrification significantly reduced the ATP content of bovine oocytes, confirming previous results in bovine^36^, human^41^, rabbit^42^, murine^23^, and porcine^43^ oocytes. The plausible reason for the decreased ΔΨm and ATP content of vitrified bovine oocytes could be that mCa^2+^ overload leads to membrane permeability transition, resulting in rupture of the mitochondrial membrane^44^. The ΔΨm and ATP content were significantly increased in the RR-treated oocytes because RR is capable of inhibiting mCa^2+^ overload and protecting mitochondrial function, as shown in Table 4.

Confirming previous reports^37, 43^, our experiment also showed that vitrification significantly increased the percentage of TUNEL-positive oocytes (Table 4), which was due to their lower ∆Ψm, since loss of ∆Ψm could release proteins such as cytochrome *c* that are located in the intermembrane space, which then activates the caspase cascade that executes the apoptotic program^45, 46^. Meanwhile, the percentage of TUNEL-positive oocytes was significantly decreased in the RR groups as RR inhibits the influx of [Ca^2+^]i to the mitochondria and maintains their ΔΨm levels (Table 4).

Similar to previous results in murine^9^, porcine^47^, ovine^48^, and bovine^36^ oocytes, our results showed the cleavage and blastocyst rates of vitrified bovine oocytes were significantly reduced after parthenogenetic activation (PA) (Supplementary Methods 1.5, Table 5), which was due to the damaged mitochondrial function of vitrified oocytes^37, 39, 43, 49^. Meanwhile, RR treatment was found to increase the cleavage and blastocyst rates of vitrified bovine oocytes in the present experiment (Table 5) due to the increase of ΔΨm and ATP content mediated by RR.

Furthermore, our results showed that BAPTA + RR treatment significantly improved the cleavage and blastocyst rates of vitrified bovine oocytes, which were similar to those of the fresh group (Table 6). According to the results of Experiments 2 and 3, one possible reason for this result is that the combined effects of BAPTA and RR can increase the incidence of normal fertilisation and protect mitochondrial functions simultaneously.

The BAX protein promotes apoptosis by enhancing the release of cytochrome *c* from mitochondria^50^, while the BCL2L1 protein prevents apoptosis by inhibiting the release of cytochrome *c* from mitochondria^51^. The CASPASE-3 protein is responsible for the breakdown of cytosolic/nuclear proteins, resulting in apoptosis^52^, while the XIAP protein inhibits apoptosis via the inhibition of the CASPASE-3 protein^53^. As shown in Fig. 5, BAPTA + RR treatment significantly increased the mRNA expression levels of the anti-apoptotic genes (*BCL2L1* and *XIAP*) and decreased the mRNA expression levels of the pro-apoptotic genes (*BAX* and *CASPASE-3*), indicating that BAPTA + RR treatment significantly decreased the blastocysts’ apoptotic index. The IFN-tau protein plays an important role in regulating implantation in bovine blastocysts^54^, and its expression level is widely used to evaluate the developmental competence of embryos^55, 56^. In the present study, the *IFN-tau* gene mRNA level (Fig. 5) and the total cell number per blastocyst were significantly increased (Table 6), indicating that treatment with BAPTA + RR significantly improved the quality of blastocysts derived from vitrified oocytes after IVF.

In conclusion, our study illustrated that vitrification, especially when DMSO was used in vitrification solution, caused Ca^2+^ release from the ER into the cytoplasm, resulting in the increased [Ca^2+^]i and mCa^2+^ levels observed in vitrified bovine oocytes. Meanwhile, combined treatment of RR and BAPTA was found capable of chelating [Ca^2+^]i and inhibiting [Ca^2+^]i influx into the mitochondria, thus preventing the premature release of CGs and protecting mitochondrial function, finally improving the fertilisation capacity and developmental ability of vitrified oocytes. Our results will help to illustrate the approach by which vitrification increases [Ca^2+^]i in vitrified oocytes and improves their fertilisation capacity and developmental ability after IVF.

## Materials and Methods

Unless otherwise indicated, all chemicals and media used in the present study were purchased from Sigma Chemical Co. (St. Louis, MO, USA), and all plasticware was obtained from Nunc-ware (Nunc; Nalge Nunc International, Roskilde, Denmark).

### Ethics statement

All experimental procedures were conducted exactly according to the guidelines for the Care and Use of Laboratory Animals issued by the Animal Ethics Committee of the Institute of Special Animal and Plant Sciences, Chinese Academy of Agricultural Sciences (Permit Number: 2014-0035). The study protocol was approved by the same committee before the start of our experiments.

### Measurement of [Ca^2+^]i, ER Ca^2+^, and mCa^2+^ levels

The [Ca^2+^]i level was detected by immunostaining as described in a previous report^57^. Briefly, oocytes were incubated in presence of 5 μM Fluo-3/AM (Invitrogen, NY, CA, USA) for 30 min at 38.5 °C, followed by three washes in Dulbecco’s phosphate-buffered saline (DPBS without Ca^2+^). Then, the oocytes were observed under an Olympus fluorescence microscope (Tokyo, Japan) equipped with a CoolSNAP HQ CCD camera (Photometrics/Roper Scientific, Inc., Tucson, AZ, USA). According to the method described by Kim *et al*.^58^, Nikon EZ-C1 FreeViewer software (Nikon, Tokyo, Japan) was used to analyse the [Ca^2+^]i fluorescence intensity of oocytes. The fluorescence pixel values within a constant area from ten different cytoplasmic regions were measured with background fluorescence values subtracted from the final values.

ER Ca^2+^ staining was performed according to the instructions of the ER Ca^2+^ detection kit (GMS10267, Genmed Scientifics Inc., Arlington, MA, USA). Briefly, oocytes were washed thrice in 100 μL cleaning medium, treated with permeable solution for 4 min at 38.5 °C, and then incubated in 100 μL staining medium for 1 h at 38.5 °C. After the oocytes were washed in 100 μL cleaning medium, they were examined under a camera-equipped fluorescence microscope, and fluorescence intensity was analysed as described above. The wavelength of excitation light was 490 nm, and the wavelength of emission light was 525 nm.

mCa^2+^ detection was performed according to the instructions of mCa^2+^ detection kits (GMS10153, Genmed Scientifics Inc., Arlington, MA, USA). The procedures for the mCa^2+^ staining and analysis were similar to those described for staining and analysis of ER Ca^2+^ levels. The wavelength of excitation light was 550 nm, and the wavelength of emission light was 590 nm.

### Analysis of CG distribution

Briefly, oocytes were treated with 0.5% (w/v) pronase at 38.5 °C for 2 min to remove ZP, fixed in 3.7% (w/v) paraformaldehyde at room temperature for 30 min, and washed thrice in 0.5% bovine serum albumin (BSA) blocking solution. After being permeabilised in 0.1% (v/v) Triton X-100 in DPBS (without Ca^2+^) for 5 min, the oocytes were washed three times in blocking solution and stained with 50 μg/mL fluorescein isothiocyanate conjugated to lens culinaris agglutinin (FITC-LCA) at 38.5 °C for 30 min. Finally, the oocytes were washed three times in DPBS (without Ca^2+^), fixed, and observed using confocal microscopy (Nikon, Tokyo, Japan). The distribution of CGs was divided into four categories according to the method described by Tian *et al*.^30^: periphery, discontinuous periphery, completed release, and homogeneous distribution.

### Determination of ΔΨm level and ATP content

JC-1 dye (Molecular Probes, Eugene, OR, USA) was used to measure the ΔΨm of the oocytes according to the procedure described by Smiley *et al*.^59^. Oocytes were incubated with 10 µg/mL JC-1 at 38.5 °C in 5% CO_2_ for 10 min, after which they were washed in DPBS (without Ca^2+^) to remove surface fluorescence, and the oocytes were observed under a confocal microscope (Nikon). Laser excitation was set at 488 nm, and emission channels of 515/30 and 650 LP were used to detect green and red fluorescence, respectively. The fluorescence intensity was quantified as described above, and ΔΨm was used as an indicator of mitochondrial function and analysed using the ratio of red to green fluorescence^34^.

A bioluminescence assay kit (ATP Bioluminescence Assay Kit HS II, Roche Diagnostics GmbH, Mannheim, Germany) was used to detect the ATP content of oocytes as described by Van Blerkom *et al*.^60^. Before analysis, oocytes were treated with 0.5% (w/v) pronase for 2 min to remove ZP. Then, 20-μL aliquots of cell lysis reagent were added to 0.5-mL centrifuge tubes containing 10 oocytes, and the ZP-free oocytes were homogenised and lysed by repeated pipetting. Meanwhile, a standard curve containing 0.01–10.0 *p*mol ATP was generated for each series of analyses. Then, 100 μL of ATP detection solution was added to wells of 96-well dishes and equilibrated for 3–5 min. Finally, standard solutions and samples (20 μL) were added to each well and luminescence was immediately measured using a luminometer (InfiniteM200, Tecan Group Ltd., Untersbergstrasse, Austria) for 10 s. The ATP content of the samples was determined from a standard curve.

### Detection of DNA fragmentation by TUNEL

A TUNEL detection kit (Roche, Indianapolis, IN, USA) was used to detect DNA fragmentation according to the manufacturer’s instructions. After three washes in DPBS without Ca^2+^, oocytes were first fixed in 4% paraformaldehyde solution for 40 min and then treated with 0.1% Triton X-100 solution for 40 min at room temperature. After being exposed to blocking solution (0.5% BSA in DPBS without Ca^2+^) at 4 °C overnight, the oocytes were stained with TUNEL solution at 38.5 °C for 1 h, then incubated in 50 µg/mL propidium iodide (PI) solution for 10 min to stain the nuclei of membrane-damaged cells. Finally, they were examined under a confocal microscope (Nikon) with the nuclei of TUNEL-positive oocytes stained yellowish green and normal oocyte nuclei stained orange-red. For the positive control, oocytes were treated with 50 µg/mL of DNase I solution for 1 h and then stained with TUNEL.

## Experimental design

### Experiment 1: Effect of vitrification on Ca^2+^ levels in bovine oocytes

Oocytes were divided into five groups: the fresh group contained untreated oocytes; the toxicity group consisted of oocytes exposed to pretreatment solution (Supplementary Methods 1.3) for 30 s, vitrification solution for 25 s, rinsed in 0.25 M sucrose solution for 1 min at 38.5 °C, followed by rinsing in 0.15 M sucrose for 5 min; the EG group consisted of oocytes exposed to 10% EG for 10 min; the DMSO group consisted of oocytes exposed to 10% DMSO for 10 min; the vitrification group consisted of oocytes that were vitrified as described in the supplementary information (Supplementary Methods 1.2, 1.3 and 1.4). The [Ca^2+^]i, ER Ca^2+^, and mCa^2+^ levels of these five groups were then analysed. The experiments were all biologically repeated three times.

### Experiment 2: Effect of BAPTA treatment on Ca^2+^ levels and the developmental ability of vitrified bovine oocytes

A stock solution of BAPTA (A1076, Sigma; 30 mM in DMSO) was diluted to the final concentration used in the experiment according to the experimental design. The medium was pre-incubated for 2 h after BAPTA addition. After *in vitro* maturation (IVM) (Supplementary Methods 1.1) for 20–22 h, cumulus-oocyte complexes (COCs) were treated with 0.1% (w/v) hyaluronidase for 1–2 min to remove cumulus cells; cultured in IVM medium (Supplementary Methods 1.1) supplemented with 5, 10, or 20 μM BAPTA for 2 h; and vitrified with vitrification solution (Supplementary Methods 1.3) containing 5, 10, or 20 μM BAPTA. Untreated fresh oocytes were used as the fresh group, and those that were vitrified without BAPTA were used as the vitrification group. Oocytes from these five groups (fresh, vitrification, 5 µM BAPTA, 10 µM BAPTA, and 20 µM BAPTA) were used to detect [Ca^2+^]i, ER Ca^2+^, and mCa^2+^ levels; CG distribution; and the fertilisation and developmental capacity of vitrified bovine oocytes after IVF (Supplementary Methods 1.6). The whole experiment was biologically repeated three times.

### Experiment 3: Effect of RR on Ca^2+^ levels and developmental potential of vitrified bovine oocytes

A stock solution of RR (R2751, Sigma; 10 mM in DPBS without Ca^2+^) was diluted to the final concentration used in the experiment according to the experimental design. The medium was pre-incubated for 2 h after RR addition. To investigate the effect of RR on Ca^2+^ levels and the developmental potential of vitrified bovine oocytes, bovine oocytes were vitrified using vitrification solution containing 0.5, 1, or 2 μM RR and incubated in IVM medium containing 0.5, 1, or 2 μM RR for 0.5 h after thawing in a CO_2_ incubator (5% CO_2_, 38.5 °C, humidified air). Untreated fresh bovine oocytes were used as the fresh group, and those that were vitrified without RR belonged to the vitrification group. The [Ca^2+^]i, mCa^2+^, ER Ca^2+^, ATP, ΔΨm, DNA fragmentation, and developmental potential after PA (Supplementary Methods 1.5) of these five groups were examined. The experiments for Ca^2+^ staining, ΔΨm, DNA fragmentation and development potential of vitrified bovine oocytes were all biologically repeated three times, and the experiment for ATP content was biologically repeated four times.

### Experiment 4: Combined effect of BAPTA and RR on the developmental ability of vitrified bovine oocytes after IVF

After IVM for 20–22 h, COCs were treated in 0.1% (w/v) hyaluronidase for 1–2 min to remove cumulus cells and cultured in IVM medium supplemented with 10 μM BAPTA for 2 h. Then, the oocytes were vitrified using vitrification solution containing 1 μM RR and 10 μM BAPTA and incubated in IVM medium containing 1 μM RR for 0.5 h after thawing. Untreated fresh bovine oocytes were used as the fresh group, and those that were vitrified without BAPTA and RR belonged to the vitrification group. The Ca^2+^ levels ([Ca^2+^]i, ER Ca^2+^, and mCa^2+^), fertilisation capacity and developmental ability (Supplementary Methods 1.6, 1.7 and 1.8) of vitrified bovine oocytes from these three groups were evaluated. The IVF experiment was biologically repeated six times, and the Ca^2+^ level analysis of oocytes, the total cell number counting and qRT-PCR experiments on blastocysts were all biologically repeated three times.

### Statistical analysis

All the results are presented as the mean ± standard error. Percentage data were previously checked for normality and homogeneity of variance followed by Duncan’s test. A statistical package (SAS Software; SAS Institute, Cary, NC, USA) was utilized to analyse one-way analysis of variance. *P* < 0.05 was considered statistically significant.

## Electronic supplementary material

SUPPLEMENTARY INFO
