# Supplementary material for: Calcium ion regulation by BAPTA-AM and ruthenium red improved the fertilisation capacity and developmental ability of vitrified bovine oocytes
Source: Sci Rep. 2017 Sep 6;7:10652. doi: 10.1038/s41598-017-10907-9 (PMC5587528; doi:10.1038/s41598-017-10907-9)
Supplement: Supplementary file 1 — SUPPLEMENTARY INFO [file 41598_2017_10907_MOESM1_ESM.pdf]

Calcium ion regulation by BAPTA-AM and ruthenium red improved the fertilisation capacity and developmental ability of vitrified bovine oocytes

Na Wang[1#], Hai-Sheng Hao[#1], Chong-Yang Li[1#], Ya-Han Zhao[1], Hao-Yu Wang[1], Chang-Liang Yan[2], Wei-Hua Du[1], Dong Wang[1], Yan Liu[1], Yun-Wei Pang[1], Hua-Bin Zhu[1], Xue-Ming Zhao[1*]

[1]Embryo Biotechnology and Reproduction Laboratory, Institute of Animal Sciences (IAS), Chinese Academy of Agricultural Sciences (CAAS), No. 2 Yuanmingyuan Western Road, Haidian District, Beijing 100193, P.R. China.

[2]Livestock and Poultry Import & Export Dept, China Animal Husbandry Group (CAHG), No. 188 West Road, South 4th Ring Road, Beijing 100070.

[*]Corresponding author: Xue-Ming Zhao: zhaoxueming@caas.cn

[#]These authors contributed equally to this work.

SUPPLEMENTARY INFORMATION

1 Supplementary Methods

1.1 *In vitro* maturation (IVM) of oocytes

For IVM, bovine ovaries collected from a local abattoir were placed in physiological saline solution containing 75 μg/mL penicillin and 50 μg/mL streptomycin and transported to the laboratory at 35 ℃ within 2 h. Cumulus-oocyte complexes (COCs) were aspirated from follicles with a diameter of 2–8 mm, and only those with at least three layers of compact cumulus cells were selected and used for IVM. Fifty COCs were cultured in 500 μL of IVM medium for 22–24 h in a CO_2 incubator (5% CO_2, 38.5 ℃, humidified air). The IVM medium contained Medium-199 (Gibco BRL, Grand Island, NY, USA), 10 mg/mL estradiol (E_2), 10 mg/mL follicle-stimulating hormone (FSH), 10% foetal bovine serum (FBS; Gibco BRL), and 10 mg/mL luteinizing hormone (LH). After IVM for 22–24 h, cumulus cells were removed from the COCs by pipetting in 0.1% (w/v) hyaluronidase for 1–2 min, and oocytes with a first polar body and uniform cytoplasm were used for subsequent experiments according to the experimental design.

1.2 Preparation of open-pulled straws (OPS)

Briefly, 0.25-mL plastic straws (IMV, L'Aigle, France) were heat-softened and pulled according to the method described by Vajta et al.[1]. The external diameter of the tips was approximately 0.23 mm, and the wall thickness was approximately 0.02 mm, as measured with a microforge.

1.3 Preparation of pretreatment and vitrification solutions

The pretreatment solution consisted of 10% (v/v) ethylene glycol (EG), 10% (v/v) dimethyl sulfoxide (DMSO) and 3 mg/mL bovine serum albumin (BSA) in Dulbecco's phosphate-buffered

saline (DPBS without Ca^{2+}). Vitrification solution consisted of 20% (v/v) EG and 20% (v/v) DMSO in FSF solution, which consisted of DPBS (without Ca^{2+}), 300 g/L Ficoll, 0.5 M sucrose, and 20% (v/v) FBS.

The warming solution was 0.15 M and 0.25 M sucrose in DPBS (without Ca^{2+}).

1.4 Vitrification and warming of oocytes

Oocytes were vitrified by being incubated in pretreatment solution for 30 s, transferred to vitrification solution for 25 s, and then loaded into OPS and vertically plunged into liquid nitrogen (LN_2).

To warm the oocytes, OPS were removed from LN_2, and the oocytes were quickly expelled and immersed in 0.25 M sucrose solution for 1 min at 38.5 ℃, followed by immersion in 0.15 M sucrose for 5 min. They were washed twice and then evaluated by observing the membrane integrity and zona pellucida (ZP)[2]. Based on the results, only surviving oocytes were used for the experiment.

1.5 Analysis of the developmental potential of oocytes by parthenogenetic activation (PA)

Oocytes were treated with 5 μM of the calcium ionophore A23187 for 5 min, then transferred into 2 mM 6-dimethylaminopurine for 6–8 h. After activation, the oocytes were placed into CR1aa medium[3] supplemented with 0.1% (w/v) BSA and cultured for 48 h. After selection of the cleaved embryos, they were counted, transferred to CR1aa medium containing 10% (v/v) FBS, and incubated for 5 days. Then, half the volume of the culture medium was replaced with fresh medium every 48 h, and the number of blastocysts was recorded on day 7.

1.6 Analysis of fertilisation and developmental capacities of oocytes by *in vitro* fertilisation

(IVF)

IVF was performed according to the method described by Brackett and Oliphant[4] with some modifications. Frozen semen was thawed at 38 ℃, then added to a 15-mL centrifuge tube containing 7 mL of Brackett and Oliphant (BO) medium supplemented with 2.5 mM caffeine and centrifuged at 1500 rpm for 5 min. After being washed twice, the supernatant was discarded and the precipitate was diluted with BO fertilisation medium containing 20 mg/mL heparin, 20 mg/mL BSA, 100 IU/mL penicillin, and 100 IU/mL streptomycin to obtain a final concentration of 5×10^6 sperms/mL. Finally, 20 μL of the sperm suspension was added to an 80-μL drop of BO fertilisation medium and incubated for 1.5 h. Then, 20–30 oocytes were transferred to the fertilisation medium and incubated for 6–8 h in a CO_2 incubator.

To analyse the fertilisation capacity of the oocytes, the putative zygotes were selected from the fertilisation drop and treated with 0.5% (w/v) pronase for 2 min to digest the ZP. Then, ZP-free zygotes were incubated in 10 μg/mL Hoechst-33342 for 10 min at 38.5 ℃. Finally, the ZP-free zygotes were washed three times, fixed on a slide, and covered with a cover slip. The nuclei of the presumed zygotes were then observed under a fluorescence microscope (Olympus, Tokyo, Japan).

To analyse the developmental capacity of oocytes after IVF, 20−30 presumed zygotes were incubated in 100-μL CR1aa droplets containing 0.1% (w/v) BSA for 2 days. Cleaved oocytes were then recorded and cultured in CR1aa medium supplemented with 10% (v/v) FBS for a further 5 days. Half of the volume of the medium was replaced with fresh medium every 48 h, and the blastocyst rates were determined on day 7.

1.7 Number of blastocyst cells

Bovine blastocysts collected on day 7 after PA or IVF were washed three times in DPBS (without

Ca^{2+}) and incubated in 10 μg/mL Hoechst-33342 for 10 min at 38.5 ℃. After being washed three times in DPBS (without Ca^{2+}), the blastocysts were fixed on a slide, covered with a cover slip, and examined under a camera-equipped fluorescence microscope to count the number of blastocyst cells.

1.8 Quantitative real-time polymerase chain reaction (qRT-PCR) of target genes in bovine blastocysts

According to the procedure described by Sun et al.[5], the Single Cell-to-CT quantitative real-time PCR kit (Life Technologies, Carlsbad, CA, USA) and the 7900HT System (Applied Biosystems, Foster City, CA, USA) were used to detect the mRNA expression levels of target genes in the bovine blastocysts. PCR was carried out in a total volume of 10 μL, which contained 2 μL of complementary DNA (cDNA), 5 μL of Fast SYBR Green Master Mix (Invitrogen), 0.2 μL (10 μM) of each primer, and 2.6 μL of water to bring the volume to 10 μL. The PCR protocol consisted of a pre-denaturation step at 95 ℃ for 10 min, followed by 40 cycles of 95 ℃ for 15 s and 60 ℃ for 1 min. The comparative Ct ($2^{-\Delta\Delta Ct}$) method[6] was calculated to represent the relative mRNA expression levels of the target genes. *B-Actin* was used as the reference gene, and the primers used for each gene are listed in Table S1.

Supplementary References

1. Vajta, G. et al. Open pulled straw (OPS) vitrification: a new way to reduce cryoinjuries of bovine ova and embryos. *Mol Reprod Dev*. **51,** 53–58 (1998).
2. Dinnyés, A., Dai, Y., Jiang, S. & Yang, X. High development rates of vitrified bovine oocytes following parthenogenetic activation, in vitro fertilization, and somatic nuclear transfer. *Biol Reprod*. **63,** 513–518 (2000).
3. Rosenkrans, CF. Jr. & First, N. L. Effect of free amino acids and vitamins on cleavage and developmental rate of bovine zygotes in vitro. *J Anim Sci*. **72,** 434–437 (1994).
4. Brackett, B. G. & Oliphant, G. Capacitation of rabbit spermatozoa in vitro. *Biol Reprod*. **12,** 260–274 (1975).
5. Sun, W. J. et al. Exogenous glutathione supplementation in culture medium improves the bovine embryo development after in vitro fertilization. *Theriogenology*. **84,** 716–723 (2015).
6. Schmittgen, T. D. & Livak, K. J. Analyzing real-time PCR data by the comparative C(T) method. *Nat Protoc*. **3,** 1101–1108 (2008).

2 Supplementary Tables

Table S1. Primer sequences of detected blastocyst genes

Gene	Primers (5′–3′)	GenBank accession number
BAX	F: TTCTGACGGCAACTTCAACTG	NM_173894.1
	R: CTCTCGAAGGAAGTCCAATGTC	
BCL2L1	F: AGGAGATGCAGGTATTGGTGA	NM_001077486.2
	R: CATTGTTCCCGTAGAGTTCCA	
CASPASE-3	F: TAAAGAAGACCATAGCAAAAGGAG	NM_001077840.1
	R: AGCACCACTGTCTGTCTCAATAC	
XIAP	F: ATACCCAAGGAACCCTGTCA	NM_001205592.1
	R: CCACCACAACAAAAGCACTG	
IFN-tau	F: CTGTCTGAGGACCACATGCTAG	AF238611.1
	R: CATCTCCTGAGGAAGACCAAA	
B-Actin	F: GGGAAATCGTCCGTGACATCA	NM_173979
	R: GATGGTGATGACCTGCCCGT	

3 Supplementary Figures

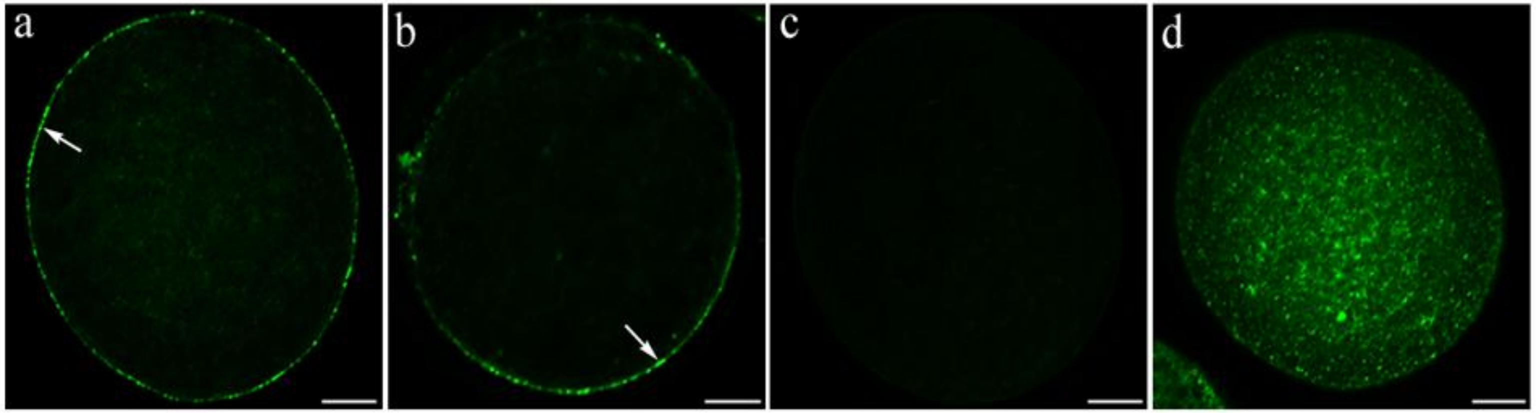

Figure S1. CG distribution in bovine oocytes stained with FITC-LCA. (a) Peripheral distribution. (b) Discontinuous peripheral distribution. (c) Completed release. (d) Homogeneous distribution (arrow indicates peripheral distribution). Scale bar = 20 µm.

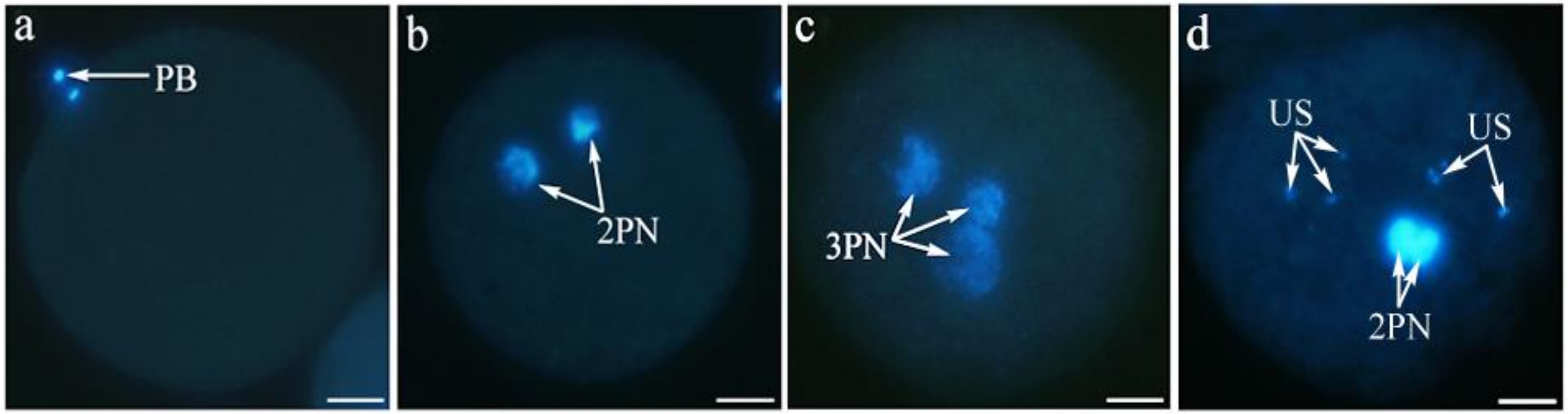

Figure S2. Fertilisation capacity analysis of bovine oocytes after IVF. (a) Unfertilised oocytes. (b) Monospermic oocyte. (c, d) Polyspermic oocytes. PB: polar body. PN: pronuclei. US: undecondensed sperm. Scale bar = 20 μm.

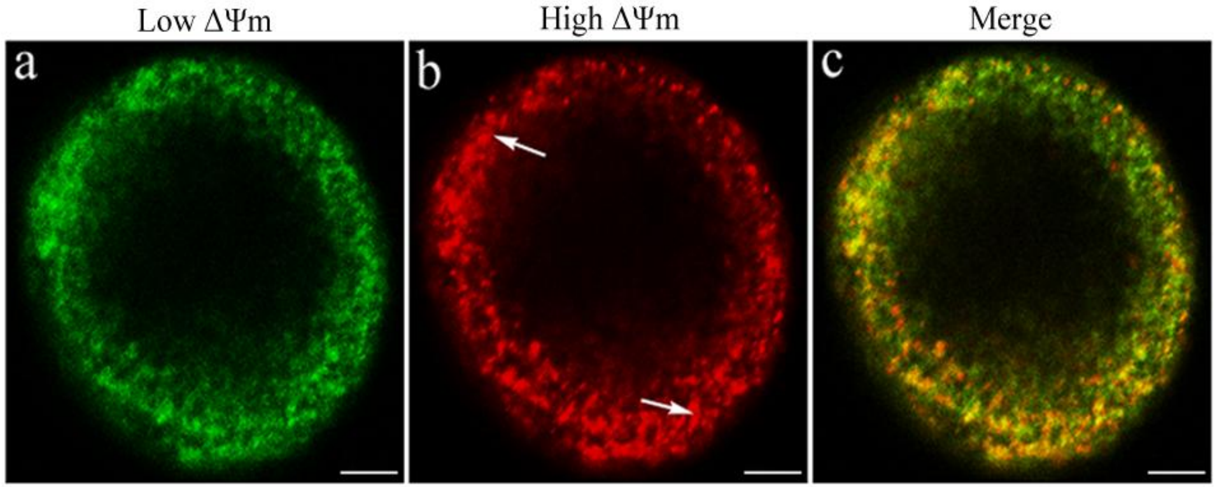

Figure S3. ΔΨm of bovine oocytes stained with JC-1. (a) Mitochondria with low ΔΨm. (b) Mitochondria with high ΔΨm. (c) Merging of images with green and red fluorescence. Arrows: mitochondrial clusters. Scale bar = 20 μm.

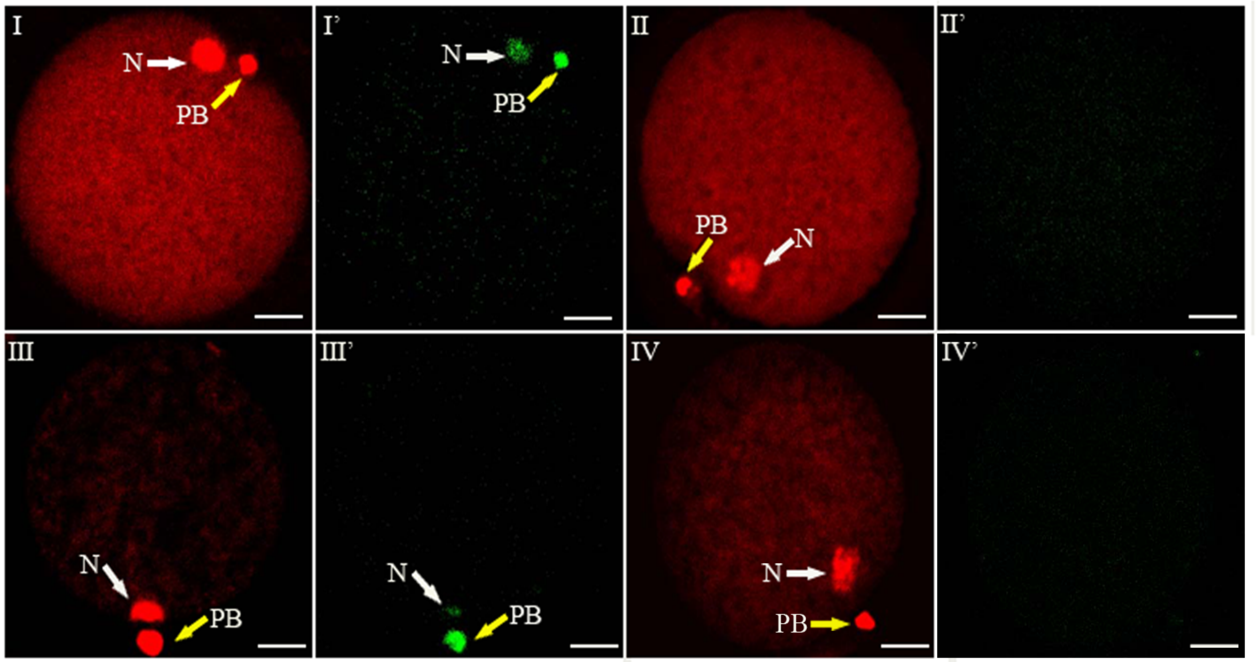

Figure S4. Oocytes stained with TUNEL. (I, I') TUNEL-positive control. (II, II') TUNEL- negative control. (III, III') TUNEL-positive oocyte. (IV, IV') Healthy oocyte. PI staining shows nuclear DNA (red) and TUNEL staining shows DNA fragmentation (green). The DNA fragmentation signal (green) was clearly visible in TUNEL-positive oocytes, but no TUNEL signal was detected in healthy oocytes. White arrowheads indicate the locations of nuclei, and yellow arrowheads indicate the locations of polar bodies. Scale bar = 20 μm.

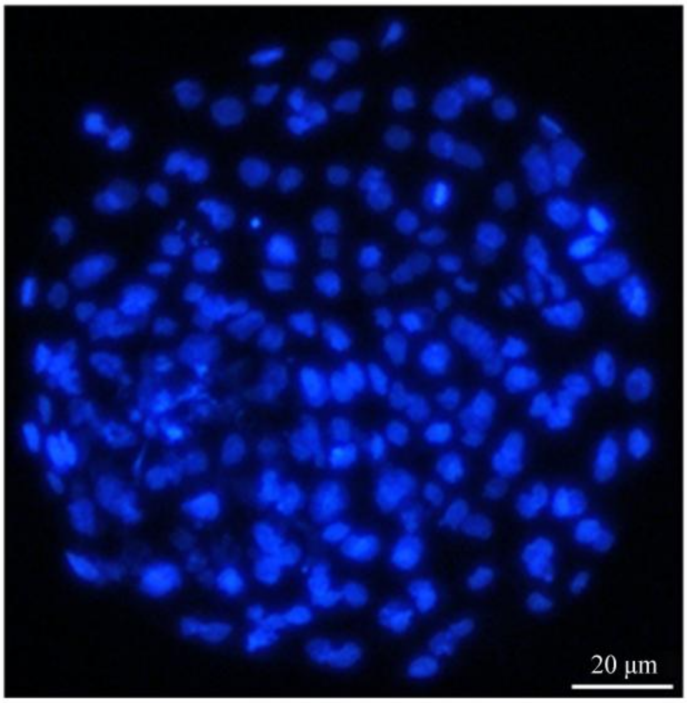

Figure S5. Blastocyst staining by Hoechst-33342. Scale bar = 20 μm.
